# Errata

**Published:** 2015-07-31

## Vol. 64, No. 18

In the report, “Controlling the Last Known Cluster of Ebola Virus Disease — Liberia, January–February 2015, the author list should read as follows: Tolbert Nyenswah^1^, Mosoka Fallah^1^, Sonpon Sieh^1^, Karsor Kollie^1^, Moses Badio^1^, Alvin Gray^1^, Priscilla Dilah^1^, Marnijina Shannon^1^, Stanley Duwor^1^, Chikwe Ihekweazu^2^, Thierry Cordier-Lasalle^2^, Shivam A. Shinde^2^, Esther Hamblion^2^, Gloria Davies-Wayne^2^, Murugan Ratnesh^2^, Christopher Dye^2^, Jonathan S. Yoder^3^, Peter McElroy^3^, Brooke Hoots^3^, Athalia Christie^3^, John Vertefeuille^3^, Sonja J. Olsen^3^, A. Scott Laney^3^, Joyce J. Neal^3^, **Sirin Yaemsiri^3^**, Thomas R. Navin^3^, Stewart Coulter^3^, Paran Pordell^3^, Terrence Lo^3^, Carl Kinkade^3^, Frank Mahoney^3^

## Vol. 64, No. 22

In the report, “Use of Serogroup B Meningococcal Vaccines in Persons Aged ≥10 Years at Increased Risk for Serogroup B Meningococcal Disease: Recommendations of the Advisory Committee on Immunization Practices, 2015,” on page 610, the fifth paragraph should read as follows: “In four clinical trials (*9–11,13*) a total of 2,557 subjects received at least 1 dose of MenB-FHbp **(*21*); no serious adverse events considered by the study investigator to be related (or possibly related) to the vaccine were reported. In three additional studies (*12*) (Pfizer, unpublished data) with a total of 7,251 subjects receiving at least 1 dose of MenB-FHbp,** four subjects reported seven serious adverse events that were considered by the study investigator to be related (or possibly related) to the vaccine.^§^ All vaccine-related serious adverse events resolved without sequelae. No increased risk for any specific serious adverse event considered to be clinically significant was identified in any of the studies. No deaths were considered to be related to MenB-FHbp. The most common solicited adverse reactions observed in the 7 days after receipt of MenB-FHbp in the clinical trials were pain at the injection site, fatigue, headache, myalgia, and chills (*21*).”

## Vol. 64, No. 28

In the report, “Launch of a Nationwide Hepatitis C Elimination Program — Georgia, April 2015,” on page 755, the second sentence should read, “**MoLHSA partnered with Gilead Sciences, a pharmaceutical manufacturer that agreed to support the program by providing an initial 5,000 courses of the antiviral medications sofosbuvir (Sovaldi), followed by 20,000 treatment courses** of ledipasvir-sofosbuvir (Harvoni) annually at no cost.

